# Challenges and Responses: A Tertiary Hospital in 2019-nCoV Epidemic

**DOI:** 10.1017/dmp.2020.93

**Published:** 2020-04-14

**Authors:** Hong Li, Zhuo Zhang, Ping Li, Hu Nie

**Affiliations:** West China Hospital, Sichuan University

**Keywords:** novel coronavirus, Emergency Service, Hospital, Emergency Preparedness, epidemic

## Abstract

The recent outbreak of coronavirus in Wuhan, China, has imposed challenges on the Chinese medical system. Not only the dramatically increasing number of infected cases and insufficient medical resources, but also the peoples’ panic throughout the whole country have made medical services extremely difficult. To respond to these challenges effectively, our hospital implemented an urgent response strategy, including human resources and medical resources preparation and re-allocation, immediate fever screening, strict patient-visiting flow management, and reasonable information communication. Our experience and response measures could provide a reference for other hospitals in the current situation.

It has been more than 1 month since the novel coronavirus outbreak in Wuhan, China, which has ravaged China and affected many countries and regions around the world. We can learn from various media about the difficulties and challenges faced by medical institutions in different regions of China during the outbreak and development of the epidemic.^1,2^ The severity of the epidemic in different regions of China is different, and the response strategies of different medical institutions may produce different results. This article intends to summarize and share our experience within 1 wk of the overall outbreak.

## NARRATIVE

Our hospital, the West China Hospital of Sichuan University (WCH, SU), is located in the Sichuan province of western China, which borders adjacent to Hubei Province. West China Hospital, a nationally recognized 4300-bed institution, is a third-level comprehensive teaching hospital, with annual outpatient visits of 5.44 million and emergency visits of approximately 280,000.

On January 7, 2020, a novel coronavirus was officially announced as the pathogen of the unknown viral pneumonia in Wuhan. On January 21, 2020, the first imported novel coronavirus pneumonia case was confirmed in Sichuan province. In contrast, on January 16, the hospital already initiated the preparation for epidemic prevention and control, including an emergency plan, organization of expert groups for medical treatment, epidemic monitoring, screening and triage, remote consultation, personnel training, personal protective equipment (PPE) reserves, and other detailed arrangements such as isolation wards extension, workflow, and patient path design. At the same time, based on our previous experience, a fever screening and triage station was set up at the emergency department (ED). All these rapid reactions benefit from the emergency response mechanism formed in disaster in the past dozen years, such as the SARS outbreak in 2003, the bird flu outbreak in 2009, and the three tremendous earthquakes in 2008, 2012, and 2017. Moreover, in 2018, the China International Emergency Medical Team (Sichuan), mainly established by our hospital, has been certified as an Emergency Medical Team by the World Health Organization.^3^ In other words, the whole hospital was preparing for a potential emergency in an orderly and routine way. At that period, most people stayed in the peace and the happiness of the approaching Lunar Chinese New Year.

However, the epidemic situation changed dramatically, and authorities implemented a cordon sanitaire in Wuhan on January 23, 2020.^4^ According to various media reports, the local epidemic situation in Wuhan was almost out of control, and the medical resources related to the prevention and control of the epidemic were extremely scarce. The public was in a state of panic. The sentiment soon spread across the country. In this situation, our hospital also faced enormous challenges, but within less than a week, it made a series of quick and effective responses.

First, emergency plans were immediately activated. All cadres who are on leave, as well as all the staff of key departments, such as the infectious disease, respiratory, emergency (ED), critical care, and experimental medicine departments, were recalled back to Chengdu to be on standby.

Second, at the same time, temporary fever triage and waiting areas were immediately set up on vacant lots close to the ED, in anticipation of the possible surge of fever cases and the potential insufficient space in the existing fever screening and triage stations.

Third, human resources were unified for allocation and management. In terms of the unified requirements of the government, a 21-member medical team was dispatched to Wuhan. Meanwhile, many specialist doctors were mobilized to receive the epidemic screening training and participate in the screening work in the fever clinic. The reception capacity of the fever clinic has been improved to cope successfully with the soaring number of fever patients.

Fourth, medical supplies were rationally allocated. Despite having prepared in advance, the country has faced a shortage of PPEs supplies since the outbreak began. The medical team sent to Wuhan took away more than half of the hospital’s protective materials, which brought some difficulties to the hospital’s epidemic prevention and control work. To solve the problem of material shortage in a short period, a strict PPEs management plan was implemented. A four-level risk classification and PPEs practice guidance were established in strict accordance with the protection guideline and some research recommendations.^5,6^ PPE resources rationing was induced in the epidemic prevention and control areas (fever clinic, triage area), as well as the regular working areas. Without any exceptional condition, a surgical mask was dispensed every 4 h, while only one N95 mask would be provided per shift in high-risk areas.

**FIGURE 1:**
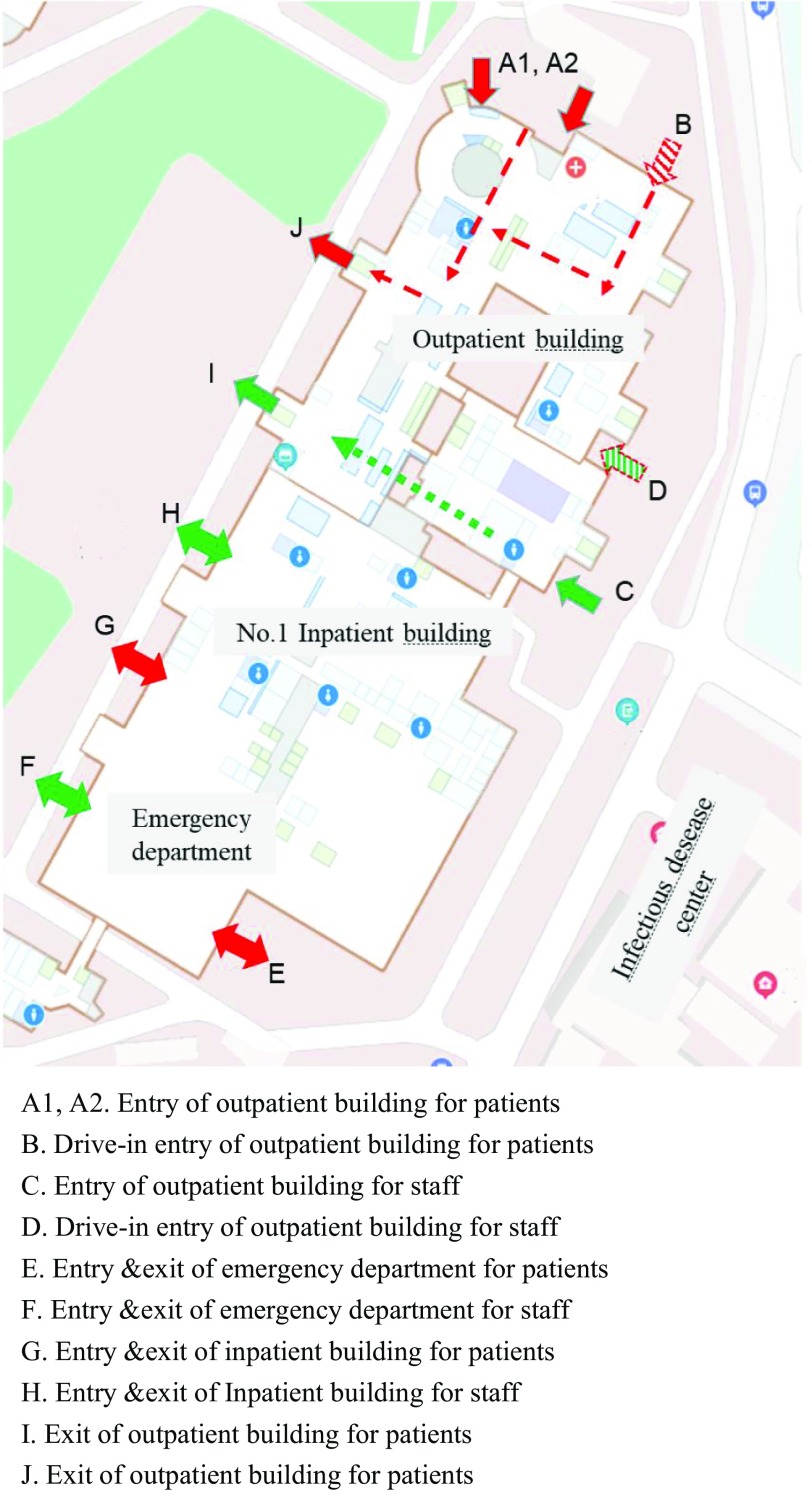
Schematic of the Single Cycle Visiting Flow Management.

Fifth, single-cycle visiting-flow management and pre-examination triage scheme were established on the whole hospital campus. Each medical building, including the outpatient building, was under closed management, with single-entry and single-exit channels. Medical staff and patients with their relatives were asked to use different entries and exits for getting in and out of the buildings (Figure 1). Volunteers were recruited at the entrance for screening, and those with abnormal temperatures or respiratory symptoms were not allowed to enter the buildings and were directed to the fever clinic. This flow management strategy helped identify one case in the first week.

Sixth, given the public’s panic about the epidemic and the possible influx of a large number of outpatient visits after the Spring Festival holiday, the hospital immediately opened a free online consultation clinic for viral-related symptoms.^7^ Using advertisements on public social media, people can directly scan a QR code by means of the mobile WeChat app to enter the online consultation platform. After completing a self-evaluation questionnaire containing disease symptoms and epidemiological history, patients can receive a free online evaluation and response from doctors within 6 h. Publicity information was released through social media, and the public was advised to reduce unnecessary hospital visits. The platform received approximately 500 online visits every day, and it helped reduce unnecessary runs on medical resources. On the first day of the outpatient service resume, the number of visits was less than 2000, which was down 90% percent from the same period last year, and the number of visits to fever clinics remained stable at approximately 100 cases per day. Therefore, the decrease in population density in the hospital helped to reduce the pressure of epidemic prevention and control significantly.

Seventh, the communication and notification of epidemic and clinical information were strengthened to stabilize the confidence of medical staff. The hospitals carried out a daily notification system for epidemic prevention and control to every department, including PPEs stock quantity, current dynamic trends of the confirmed and suspected cases in the hospital, and the daily progression of the dispatched medical team, and so on. Timely and transparent release of information helped stabilize emotions.

## DISCUSSION

Infectious diseases have become a major challenge to human society in this century. Emerging infectious diseases caused more frequent and complex epidemics in the past 2 centuries, such as SARS, Zika, MERS, and COVID-19. Global health systems are still under-prepared for any significant outbreaks, even with considerable progress.^8,9^

The recent outbreak of coronavirus in Wuhan, China, has imposed challenges on the Chinese medical system. The sharp increase in the number of COVID-19 patients and the shortage of medical supplies strained the medical service system. Public panic throughout the whole country and the following surge in hospitals almost collapsed the system in some areas. Although the epidemic situation varies from region to region, timely and reasonable preparation will contribute to effective medical responses and epidemic outbreak control.

The COVID-19 coronavirus seems to be very contagious and spreading faster and broader than other previous viruses.^10^ As happened in Wuhan and other cities around the world, once a regional outbreak occurs, a “bank run” on medical resources and the following medical system breakdown may be inevitable as numbers of patients surge. Implementation of an emergency response strategy preemptively could delay the growth and limit the size of the epidemic.^11^ To cope with this particular and possible future mass casualty incidents, every health facility should do its best to prepare for the ongoing pandemic.

Medical staff and workforce recruiting, medical resources preparation and re-allocation, and urgent expansion of essential medical spaces should be the critical emergency response strategy for every hospital and may provide essential support for epidemic prevention and control. Active and rational patient flow management in advance, such as moving line re-design and control, pre-examination triage, and remote online medical service, will help to reduce the risk of patients surge and nosocomial infections. According to our experience, all of these response strategies helped to reduce the impact on the medical system.

Through the implementation of the above measures, the hospital’s epidemic prevention and control and various clinical work have been carried out in an orderly manner. At the time of writing, the number of outpatient visits and inpatient admissions was stable and reasonably controlled. We hope that these measures will continue to help effectively control the epidemic, and our experiences will provide a reference for other medical institutions.
